# Quantitative Validation of a GNSS Campaign Planning Model for Kinematic Surveys: A Comparison of GPS and GPS/GLONASS Configurations

**DOI:** 10.3390/s26185864

**Published:** 2026-09-16

**Authors:** Mariusz Specht, Sławomir Figiel, Cezary Specht

**Affiliations:** Department of Transport, Gdynia Maritime University, Morska 81-87, 81-225 Gdynia, Poland; slawomir@figiel.xyz (S.F.); c.specht@wn.umg.edu.pl (C.S.)

**Keywords:** GNSS, campaign planning, GPS, GLONASS, PDOP, model validation, kinematic surveys

## Abstract

**Highlights:**

**What are the main findings?**

- In the field observations, the GPS/GLONASS configuration reduced mean PDOP by approximately 30% and increased satellite availability by 81–86% relative to the GPS-only configuration.
- The mean absolute model-observation PDOP difference decreased by 58.0–68.4%, with the greatest improvement observed in dense urban environments.

**What are the implications of the main findings?**

- The GPS/GLONASS configuration improves model-observation agreement, with the magnitude of the improvement depending on local observation conditions.
- The proposed methodology can support the planning of mobile GNSS measurement campaigns in sensor-based environmental monitoring applications.

**Abstract:**

Reliable georeferencing is a fundamental requirement for modern sensor-based environmental monitoring systems employing mobile sensing platforms. This study presents a quantitative validation of a GNSS campaign planning model for kinematic surveys and evaluates how extending a single-constellation GPS configuration to a dual-constellation GPS/GLONASS configuration affects model-observation agreement under real-world conditions. The analysis was based on a kinematic measurement campaign conducted along an 18.4 km route in the Tricity metropolitan area (Gdańsk-Sopot-Gdynia), Poland. Two GNSS receivers operating at 1 Hz were used simultaneously, providing GPS and GPS/GLONASS observations under comparable kinematic and environmental conditions. Model predictions derived from DSM and DTM data were compared with field observations in terms of PDOP, satellite visibility, and model-observation agreement. The results demonstrate that the GPS/GLONASS configuration substantially improved satellite constellation geometry relative to the GPS-only configuration. Mean PDOP values decreased by approximately 30%, while the number of visible satellites increased by 81–86%. More importantly, the GPS/GLONASS configuration improved model-observation agreement. The mean absolute model-observation PDOP difference was reduced by 58.0% for the entire route, 66.7% under favourable observation conditions, and 68.4% under challenging observation conditions. The greatest improvements in model-observation agreement were achieved in dense urban environments affected by signal obstructions. These results demonstrate that constellation configuration influences not only satellite constellation geometry but also the agreement between trajectory-based campaign-planning predictions and real kinematic observations. The proposed methodology may also support the planning of sensor-based environmental monitoring campaigns requiring favourable GNSS observation conditions along measurement trajectories.

## 1. Introduction

### 1.1. Sensor-Based Environmental Monitoring and the Role of GNSS

Sensor-based environmental monitoring has become an essential component of sustainable environmental management, supporting applications such as coastal monitoring, water quality assessment, environmental mapping, ecosystem observation, infrastructure inspection, and mobile mapping [1,2,3,4,5]. The increasing use of mobile sensing platforms, including unmanned aerial vehicles (UAVs) and unmanned surface vehicles (USVs), enables efficient acquisition of spatially distributed environmental data over large areas [2,3,5,6]. Equipped with multiple onboard sensors, these platforms constitute mobile sensing systems requiring precise positioning to ensure accurate georeferencing and integration of heterogeneous environmental observations [7,8,9,10,11].

Modern environmental monitoring systems increasingly rely on multisensor platforms integrating optical, laser, radar, hydrographic, and navigation sensors [7,8,9,10]. Such systems enable the simultaneous acquisition of complementary spatial information, improving the accuracy, completeness, and reliability of environmental observations [1,5,7,8,9]. However, regardless of the sensor configuration, all collected data require precise spatial referencing to ensure their interoperability and seamless integration within geographic information systems (GIS). Consequently, the overall quality of environmental products derived from multisensor observations depends directly on the accuracy of positioning and spatial referencing [9,10,11].

Among the technologies supporting accurate georeferencing, Global Navigation Satellite Systems (GNSS) have become one of the fundamental components of mobile sensing platforms [9,10,11]. They provide the positioning information required for synchronising measurements acquired by onboard sensors and enable the integration of heterogeneous datasets collected from different platforms, including UAVs, USVs, and terrestrial mobile mapping systems [7,8,9,10,11,12]. Taken together, previous studies demonstrate that accurate georeferencing has become a prerequisite for reliable sensor-based environmental monitoring applications [1,5,7,8,9,10,11,12].

GNSS technology has become fundamental to geodetic, navigation, and transport applications [13,14,15,16]. Positioning accuracy depends not only on the quality of GNSS observations but also on the geometry of the satellite constellation relative to the receiver. This effect is described by the dilution of precision (DOP), particularly the position dilution of precision (PDOP), which quantifies the influence of satellite constellation geometry on 3D positioning accuracy and is one of the key parameters used in GNSS campaign planning [17,18].

For this reason, GNSS campaign planning is an essential stage in the preparation of measurements, enabling the identification of time intervals with the most favourable observation conditions [19,20]. However, existing approaches often evaluate GNSS observation conditions at predefined stationary locations [21], which is insufficient for mobile measurement campaigns along linear infrastructure, such as roads, railways, or tram lines.

In such applications, GNSS signal reception conditions change dynamically as the receiver moves, particularly in urban environments, where buildings, infrastructure, and vegetation limit satellite visibility and degrade satellite constellation geometry. Additionally, multipath effects and non-line-of-sight (NLOS) signals may significantly degrade observation quality [22,23,24,25,26]. As a result, both favourable and highly degraded observation conditions may occur along a single trajectory.

This indicates that the evaluation of GNSS campaign planning requires approaches that account for the spatial variability of observation conditions and the impact of environmental obstacles on GNSS signal propagation, particularly in urban environments, where observation conditions may change significantly over short distances.

### 1.2. Influence of Satellite Constellation Geometry and Number of Visible Satellites on PDOP

One of the key factors affecting GNSS positioning accuracy is satellite constellation geometry relative to the receiver. PDOP quantifies the influence of satellite constellation geometry on positioning accuracy. PDOP values decrease as satellites become more evenly distributed across the sky and as their number increases [17,18,27,28].

An increase in the number of available satellites generally improves constellation geometry and leads to lower PDOP values, resulting in more favourable conditions for GNSS positioning [29,30,31]. Consequently, the number of visible satellites and PDOP are commonly used as key indicators of observation conditions in GNSS campaign planning [32,33].

These parameters are particularly important in environments with limited sky visibility, where satellite availability and geometry may be significantly degraded. Under such conditions, multi-constellation (multi-GNSS) solutions can increase the number of visible satellites, improve constellation geometry, and consequently reduce PDOP values.

### 1.3. Limitations of Analyses Based on a Single Constellation

In GNSS campaign planning, multi-constellation solutions, including the Global Positioning System (GPS), GLONASS, Galileo, and BeiDou, are increasingly used [34,35]. However, many existing studies are still based on simplified approaches that do not provide a detailed comparison of different GNSS solutions under varying observation conditions, nor do they assess their implications for GNSS campaign planning results [36,37].

In particular, there is limited evidence on how constellation configuration affects the agreement between trajectory-based campaign-planning predictions and real kinematic observations under spatially varying GNSS observation conditions. This issue is especially important in environments with limited sky visibility, such as urban areas, where GNSS signals are frequently obstructed by buildings and vegetation.

Under these conditions, additional constellations can significantly improve satellite availability and constellation geometry [38,39]. Nevertheless, their influence on the agreement between campaign-planning predictions and real observations along measurement trajectories has not been systematically quantified.

### 1.4. Previous Validation of the Model for the GPS Configuration

In response to the identified limitations in previous studies, a GNSS campaign planning model was developed for measurements conducted in motion along linear features. The model accounts for the measurement trajectory and the influence of environmental obstacles, modelled using a Digital Surface Model (DSM) and a Digital Terrain Model (DTM) [19,20].

In a preceding stage of the research, the model was quantitatively validated using data from a field measurement campaign [40]. That analysis considered a GPS-only configuration and focused on model-observation agreement in terms of PDOP and satellite availability, as well as on identifying the principal sources of discrepancies between model predictions and field observations. The results showed that the model reproduced the general variability of observation conditions along the measurement route, while the remaining discrepancies were associated mainly with the representation of environmental obstacles, particularly vegetation, in the DSM. The GPS-only scope of that validation also limited the assessment of the model in the context of contemporary multi-GNSS solutions. Similar limitations in the representation of environmental obstacles have been reported in studies on GNSS simulation in urban environments [41,42]. Consequently, although the principal sources of discrepancies for the GPS-only configuration were identified, it remained unknown whether constellation configuration itself constitutes an additional factor affecting model-observation agreement.

### 1.5. Research Gap: Influence of Constellation Configuration on Model-Observation Agreement

Despite the growing use of GNSS-supported sensor systems for environmental monitoring and mobile mapping, considerably less attention has been given to the agreement between trajectory-based GNSS campaign-planning predictions and real kinematic observations under spatially varying GNSS observation conditions. This issue is particularly relevant in urban environments, where satellite visibility and constellation geometry may change substantially over short distances because of local obstructions.

The inclusion of additional constellations, such as GLONASS, increases the number of observed satellites and improves satellite constellation geometry, potentially affecting not only positioning performance but also the reliability of GNSS campaign planning results [29,30,36,38]. To the authors’ knowledge, no previous study has provided a quantitative, trajectory-based comparison of GPS and GPS/GLONASS configurations for GNSS campaign planning in kinematic surveys together with validation against real-world measurements and an assessment of their influence on model-observation agreement. The scientific contribution of this study is not the demonstration that adding a second GNSS constellation increases satellite availability, but the quantitative assessment of how the transition from GPS-only to GPS/GLONASS affects model-observation agreement in GNSS campaign planning for kinematic surveys under real-world urban conditions. Furthermore, this effect is evaluated for the entire measurement route and for representative favourable and challenging observation environments, enabling assessment of whether its magnitude depends on local GNSS visibility conditions.

The proposed approach also provides a methodological framework for future analyses involving full multi-GNSS solutions, including GPS, GLONASS, Galileo, and BeiDou. To further clarify the distinction between the preceding GPS-only validation stage and the present study, Table 1 compares their objectives, scope, methodology, and outcomes.

As shown in Table 1, the present study addresses a different validation question from the preceding GPS-only study. The earlier stage focused on validating the campaign-planning model and identifying the principal sources of discrepancies between model predictions and GPS observations. In contrast, the present study treats constellation configuration as an additional experimental factor and evaluates how extending the configuration from GPS-only to GPS/GLONASS affects model-observation agreement. This effect is analysed for the entire measurement route and for representative favourable and challenging observation environments.

### 1.6. Aim of the Study

Reliable planning of GNSS observations is an important prerequisite for mobile sensor systems that require accurate georeferencing of environmental measurements. The aim of this study is to quantitatively determine how extending a single-constellation GPS configuration to a dual-constellation GPS/GLONASS configuration affects the agreement between a trajectory-based GNSS campaign planning model and real kinematic observations under spatially varying GNSS observation conditions.

To achieve this aim, the following specific objectives were formulated:

1. To quantify the effect of extending the GPS-only configuration to GPS/GLONASS on model-observation agreement using PDOP and satellite availability as quantitative indicators of satellite constellation geometry;
2. To compare the magnitude and variability of model-observation discrepancies for the GPS and GPS/GLONASS configurations along the entire measurement route;
3. To determine whether the influence of constellation configuration on model-observation agreement depends on local observation conditions by analysing representative favourable and challenging environments.

Accordingly, PDOP and satellite availability are used as quantitative indicators supporting the model-validation analysis rather than as stand-alone evidence of the benefits of an additional GNSS constellation.

### 1.7. Structure of the Paper

The paper is organised as follows. Section 2 describes the study area, measurement system, GNSS campaign planning model, data pre-processing procedure, comparative methodology, and statistical analysis. Section 3 presents the results of the comparative analysis. Section 4 discusses the findings and evaluates the influence of the GPS/GLONASS configuration on model-observation agreement. Section 5 summarises the main conclusions and outlines directions for future research.

## 2. Materials and Methods

### 2.1. Study Area and Measurement Route

**Figure 1:**
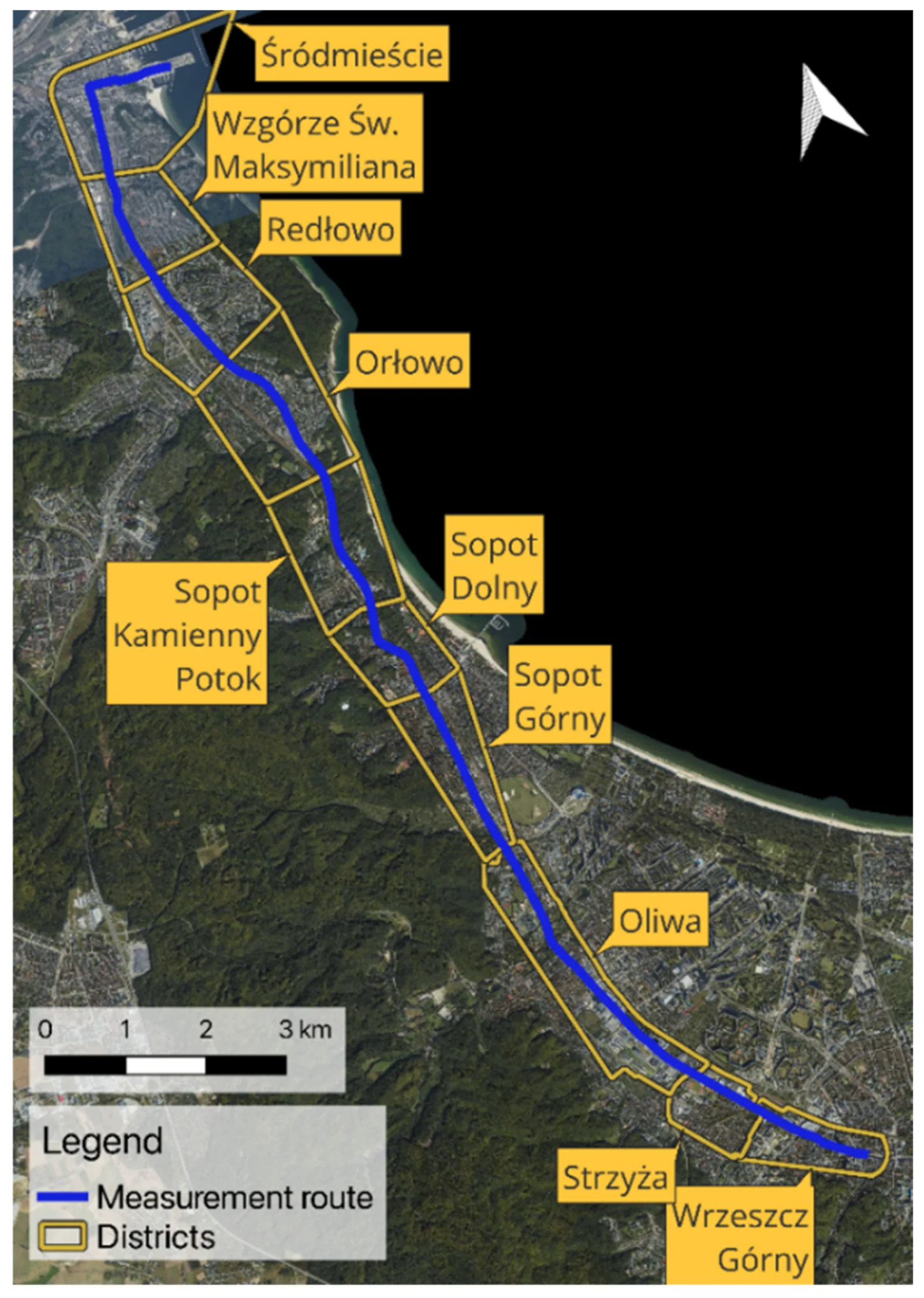
Location of the study area and the measurement route used for evaluating the GNSS campaign planning model.

The study was conducted along a main transport corridor within the Tricity metropolitan area (Gdańsk-Sopot-Gdynia) in northern Poland (Figure 1). The measurement route, with a total length of 18.4 km, included sections characterised by varying observation conditions, including areas with good sky visibility and segments of dense urban development that limited GNSS signal availability. Along the route, multi-storey buildings and land cover elements, including trees and roadside vegetation, were present.

The variability of local environmental conditions affected GNSS signal availability and satellite constellation geometry. In areas of dense development and tall vegetation, GNSS signal obstruction and multipath effects were more pronounced. The measurement campaign was conducted on 10 September 2024 during night-time hours, which reduced the effects of traffic and facilitated the maintenance of a relatively constant vehicle speed.

### 2.2. Measurement System and GNSS Campaign Implementation

The measurement vehicle was equipped with two GNSS receivers (Garmin 19x HVS), mounted on the vehicle roof at a distance of 1 m from each other, along a line perpendicular to the longitudinal axis of the vehicle, at a height of 1.7 m above ground level. One of the receivers recorded signals exclusively from GPS, whereas the other received signals from both GPS and GLONASS, enabling their comparison under comparable measurement conditions. The simultaneous use of two identical receivers enabled both GNSS configurations to be evaluated during the same survey run, limiting the influence of changes in satellite constellation geometry and local GNSS signal reception conditions on the comparison results. The identical hardware configuration also reduced differences attributable to receiver characteristics. Using a single receiver sequentially in different GNSS configurations would require separate survey runs and would therefore introduce temporal changes in satellite constellation geometry and local GNSS signal reception conditions into the comparison.

**Figure 2:**
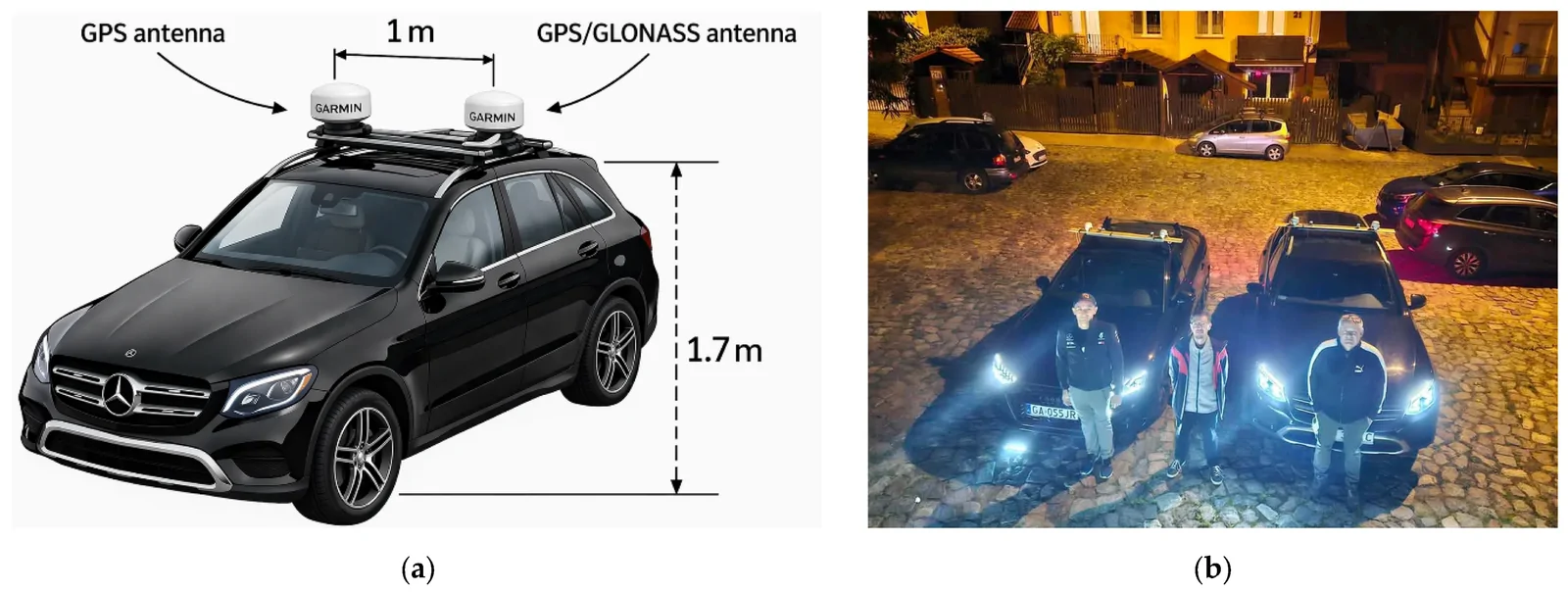
Configuration of the GNSS measurement system: arrangement of GNSS antennas on the vehicle roof (**a**); field measurement campaign conducted under real-world conditions (**b**).

GNSS data were recorded at 1 Hz as National Marine Electronics Association (NMEA) messages of the following types: GGA, GSA, GSV, and RME. These messages contained, among other information, PDOP values, the number of visible satellites (*N*_sat_), and satellite azimuth and elevation angles. An elevation mask of 10° was applied in order to reduce the influence of low-elevation satellites, which are more susceptible to signal obstruction and multipath effects. The measurement campaign was carried out during a drive along the route at an average speed of approximately 50 km/h, providing an approximately uniform spatial sampling interval between consecutive observations. The measurements were conducted in accordance with a predefined schedule established at the planning stage. In total, 1665 measurement epochs were recorded for the GPS configuration and 1666 epochs for the GPS/GLONASS configuration, enabling comparative analyses of the two GNSS configurations. The configuration of the measurement system is shown in Figure 2.

### 2.3. GNSS Campaign Planning Model

The GNSS campaign planning model used in this study was originally developed and described in previous publications [19,20]. For completeness, the principal computational assumptions and procedures relevant to the present GPS and GPS/GLONASS comparison are described below. The model inputs comprise the measurement trajectory, campaign date and time window, vehicle speed, recording interval, antenna height, elevation mask, satellite orbital data, GNSS configuration, DSM, and DTM.

The model operates before the field campaign, predicting satellite geometry and visibility for successive positions along a predefined measurement trajectory. In contrast, GNSS observations describe the conditions actually encountered during the field campaign. By combining satellite orbital information with DSM/DTM-based obstruction modelling, the model enables favourable measurement periods and potentially problematic route sections to be identified before data acquisition. Field GNSS observations are therefore used in the present study to validate the model predictions.

The model enables the determination of a measurement schedule that optimises satellite constellation geometry, represented by PDOP values, for GNSS measurements conducted in motion along a linear feature. The model accounts for the influence of environmental obstacles on GNSS signal availability through the use of DSM and DTM, from which satellite visibility and PDOP values are determined for successive epochs. For each receiver position along the trajectory, the model determines satellite positions based on the orbital data. Satellite positions are transformed into a local topocentric coordinate system centred at the receiver position, in which their azimuth and elevation angles are determined. The calculations are performed separately for the GPS and GPS/GLONASS configurations.

For the computations, elevation data obtained from the Polish Geoportal operated by the Head Office of Geodesy and Cartography were used. The applied DSM and DTM, with a spatial resolution of 0.5 m, were referenced to the Polish implementation of the European Vertical Reference Frame 2007 in the normal height system (PL-EVRF2007-NH) and were acquired in 2022.

Satellite orbital data for GPS were obtained in YUMA almanac format from the CelesTrak portal and corresponded to GPS week 2329 (Time of Applicability, ToA = 589,824 s). Orbital data for GLONASS satellites were obtained from the National Aeronautics and Space Administration (NASA) Crustal Dynamics Data Information System (CDDIS) archive for day 254 of 2024. The GLONASS data were provided in GLONASS NAV DATA format, version 2.01, as an International GNSS Service (IGS) broadcast ephemeris file (brdc2540.24g). Thus, the GPS calculations used almanac data, whereas the GLONASS calculations used broadcast ephemerides; no precise final, rapid, ultra-rapid, or real-time orbit products were used. These data were used to determine satellite positions and the corresponding azimuth and elevation angles used in satellite visibility and PDOP calculations. The calculated azimuth and elevation angles for GPS satellites were verified against the corresponding values recorded in NMEA GSV messages. For most analysed GPS satellites, the mean azimuth and elevation differences remained within ±1°. Inaccuracies in the orbital data may affect the calculated satellite azimuth and elevation angles and may consequently influence satellite visibility classification and PDOP values. The purpose of the orbital data in the present model is to reproduce satellite constellation geometry for campaign-planning purposes rather than to perform precise point positioning. Their influence is therefore primarily associated with possible changes in predicted azimuth and elevation angles, satellite visibility classification, and consequently PDOP.

For each position along the trajectory, satellite visibility is determined using line-of-sight (LOS) analysis between the receiver antenna and each satellite, taking into account terrain obstructions and the adopted elevation mask. The elevation angle of each obstacle located along the LOS is compared with the satellite elevation angle. If the obstacle elevation angle exceeds the satellite elevation angle, the satellite is classified as obstructed. Satellites below the adopted 10° elevation mask are also excluded from the calculations. Only buildings were retained as obstacles in the DSM, while non-building above-ground objects were removed to reduce the influence of vegetation-related representation errors on satellite visibility modelling.

On this basis, the number of available satellites and the PDOP value are calculated, with PDOP adopted as the main criterion for assessing satellite constellation geometry. An observation geometry matrix is constructed from the azimuth and elevation angles of the visible satellites. All satellites classified as visible are assigned equal weight in the PDOP computation, with no elevation-dependent or signal-quality-dependent weighting applied. The corresponding covariance matrix is then derived from the observation geometry matrix and used to calculate PDOP. The calculations are performed independently for the GPS and GPS/GLONASS configurations.

In this study, the model was used to determine the measurement schedule for the campaign. *N*_sat_ and PDOP were calculated for successive epochs throughout the analysed time interval. Based on these values, the campaign schedule was determined, taking into account the adopted PDOP threshold and constraints on the minimum stop duration and the minimum distance between consecutive stops. The resulting predictions were then compared with the field observations, enabling assessment of model-observation agreement under real-world conditions. The model configuration and input parameters adopted in this study are presented in Table 2 to ensure transparency and reproducibility of the planning procedure.

For the adopted settings, a campaign schedule was determined for the time interval from 22:45 to 23:37 (52 min). The schedule included two vehicle stops: in Gdańsk, near the University of Gdańsk (25 min), and in Sopot (5 min), as determined by the optimisation procedure implemented in the planning model.

### 2.4. Pre-Processing of Measurement Data

**Figure 3:**
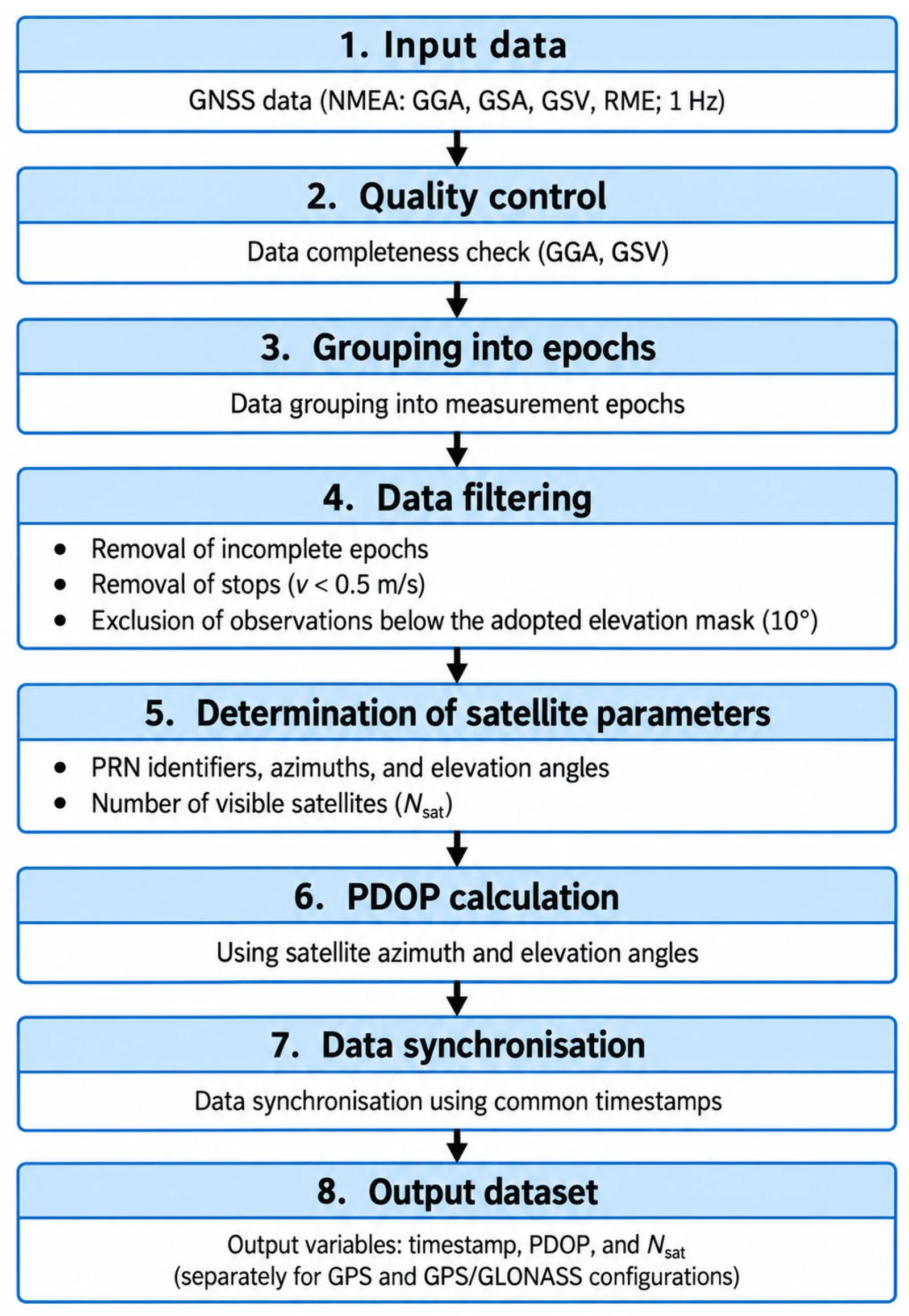
Workflow of GNSS data pre-processing applied in the comparative analysis.

GNSS measurement data recorded by both receivers were stored as NMEA messages (GGA, GSA, GSV, RME) at a 1 s interval. Pre-processing included quality control, filtering, synchronisation with modelled data, and preparation of the dataset for further comparative analysis, taking into account two GNSS configurations: GPS and GPS/GLONASS (Figure 3).

In the first stage, data completeness was verified, and the observations were grouped into measurement epochs. The time of each epoch was determined based on the timestamp in the GGA message and used for synchronisation with the modelled data.

In the next stage, measurement data were filtered. Incomplete epochs (missing GGA or GSV messages) and epochs corresponding to stops not related to the implementation of the measurement campaign schedule were excluded from the analysis. Epochs were classified as stops if the instantaneous vehicle speed was lower than 0.5 m/s, consistent with the campaign planning assumptions. Additionally, observations of satellites with elevation angles below the adopted elevation mask (10°) were removed.

Based on GSV messages, satellite Pseudo-Random Noise (PRN) identifiers, azimuths, and elevation angles were extracted. On this basis, *N*_sat_ was determined for each epoch. The PDOP value was calculated independently of the values reported by the GNSS receiver, using the standard DOP formulation based on the geometry matrix derived from satellite azimuth and elevation angles. This approach was adopted to ensure methodological consistency with the GNSS campaign planning model, which determines PDOP from satellite constellation geometry. The internal satellite-selection and weighting procedures used by the receiver to derive the reported PDOP values were not available from the recorded NMEA data. Using receiver-reported PDOP directly would therefore introduce an unknown receiver-specific processing component into the comparison, whereas independent recalculation ensured that modelled and measurement-derived PDOP values were obtained using the same geometric formulation. PDOP calculations were performed separately for the GPS and GPS/GLONASS configurations.

The synchronisation of measurement and modelled data was carried out using common timestamps. Due to differences between the actual and modelled trajectories, resulting from variations in vehicle speed and trajectory deviations, direct point-to-point matching was not applied. Instead, the comparison was based on a segment-based matching procedure described in Section 2.5.

As a result of the pre-processing steps, separate datasets were obtained for the GPS and GPS/GLONASS configurations, containing timestamps, PDOP values, and *N*_sat_ values for each retained measurement epoch. These datasets formed the basis for the subsequent comparison with the modelled data.

### 2.5. Comparative Procedure

For comparative purposes, the planning model was run for two GNSS configurations using the same date and time interval as the field measurement campaign. In the first configuration, only GPS was considered, whereas the second configuration included GPS and GLONASS. In the DSM, non-building above-ground objects were removed because their representation, particularly that of vegetation, may introduce discrepancies in LOS-based satellite visibility modelling.

The basic unit of comparison was defined as a trajectory segment rather than a single measurement epoch. Accordingly, *N* denotes the number of trajectory segments retained for analysis after spatial matching and application of the adopted quality criteria. Due to the lack of full agreement between the modelled and actual trajectories, resulting from differences in vehicle speed, minor deviations in the vehicle path, and necessary stops, direct comparison of parameters at identical positions was not possible. Therefore, a spatial data matching procedure was applied to enable direct comparison of modelled and observed data. No inertial measurement unit (IMU) data were used in the present analysis; trajectory matching and segment assignment were based exclusively on GNSS-derived positions.

The modelled trajectory was divided into equal segments, the length of which corresponded to the product of the assumed vehicle speed and the GNSS data recording interval. For the analysed campaign, the segment length was 13.89 m, corresponding to the distance travelled in one second at the assumed vehicle speed of 50 km/h. Accordingly, one measurement epoch would be expected per modelled trajectory segment under nominal conditions.

The recorded positions and modelled epochs were assigned to their corresponding trajectory segments. Several observation epochs could be assigned to a single segment because of slight differences between the actual vehicle speed and trajectory and the values assumed in the GNSS campaign planning model. The minimum observed PDOP within each segment was selected in accordance with the model design, whose purpose is to identify the most favourable observation conditions along the planned route. This value therefore represents the best satellite constellation geometry within a given segment rather than typical observation conditions. Mean or median PDOP values, as well as the observation closest to the segment centre, would characterise different aspects of the measurements and would not directly correspond to the decision criterion implemented in the campaign planning model.

Before the segment-based comparison, measurement epochs that did not satisfy the adopted geometric quality criteria were excluded. For each GNSS configuration, 24 measurement epochs were removed: for 12 epochs, the model was unable to determine at least four visible satellites, whereas for a further 12 epochs the modelled PDOP exceeded the predefined threshold of 7. This corresponded to approximately 1.4% of the recorded epochs for both the GPS and GPS/GLONASS configurations. The PDOP threshold of 7 represented the maximum acceptable value for conditions considered suitable for measurement execution and was established before the model-observation comparison. The values of *N* reported in the subsequent statistical analyses therefore refer to trajectory segments retained after spatial matching and application of the adopted quality criteria, rather than to the original number of recorded measurement epochs.

The trajectory segments were assigned to individual districts of the Tricity area based on their spatial location along the measurement route, and descriptive statistics of PDOP and *N*_sat_ were calculated for both modelled and observed data. These statistics were also determined for the entire route. The observed PDOP and *N*_sat_ values were subtracted from the corresponding modelled values, enabling a quantitative assessment of model-observation agreement.

Two types of differences were analysed. The first type was used to assess model-observation agreement for each GNSS configuration, whereas the second type enabled direct comparison of how well each configuration represented the observation conditions.

The first type comprised differences between modelled and observed values, defined as:

(1) $$\Delta {\mathrm{PDOP}}^{\mathrm{GPS}}={\mathrm{PDOP}}_{\mathrm{mod}}^{\mathrm{GPS}}-{\mathrm{PDOP}}_{\mathrm{obs}}^{\mathrm{GPS}}$$

(2) $$\Delta N_{\mathrm{sat}}^{\mathrm{GPS}}=N_{\mathrm{sat},\mathrm{mod}}^{\mathrm{GPS}}-N_{\mathrm{sat},\mathrm{obs}}^{\mathrm{GPS}}$$

(3) $$\Delta {\mathrm{PDOP}}^{\mathrm{GPS}/\mathrm{GLONASS}}={\mathrm{PDOP}}_{\mathrm{mod}}^{\mathrm{GPS}/\mathrm{GLONASS}}-{\mathrm{PDOP}}_{\mathrm{obs}}^{\mathrm{GPS}/\mathrm{GLONASS}}$$

(4) $$\Delta N_{\mathrm{sat}}^{\mathrm{GPS}/\mathrm{GLONASS}}=N_{\mathrm{sat},\mathrm{mod}}^{\mathrm{GPS}/\mathrm{GLONASS}}-N_{\mathrm{sat},\mathrm{obs}}^{\mathrm{GPS}/\mathrm{GLONASS}}$$

where

PDOP_mod_, PDOP_obs_—PDOP values from the model and observations, respectively,
*N*_sat,mod_, *N*_sat,obs_—number of visible satellites in the model and observations, respectively,
GPS and GPS/GLONASS denote the applied GNSS configurations,
ΔPDOP and Δ*N*_sat_ denote differences between modelled and observed values.

Positive values indicate overestimation by the model, whereas negative values indicate underestimation. Lower absolute values indicate better model-observation agreement.

The second type of difference was an indicator comparing the absolute differences between the model and observations for the GPS and GPS/GLONASS configurations, defined as:

(5) $$\Delta \left|\mathrm{PDOP}\right|=\left|{\Delta \mathrm{PDOP}}^{\mathrm{GPS}/\mathrm{GLONASS}}\right|-\left|{\Delta \mathrm{PDOP}}^{\mathrm{GPS}}\right|$$

Negative values of Δ|PDOP| indicate better agreement of the GPS/GLONASS configuration with the measurement data, whereas positive values indicate better agreement of the GPS configuration. Values equal to zero indicate identical agreement for both configurations. Selected districts representing the most favourable and the most challenging observation conditions were analysed in detail, together with the entire route, enabling the assessment of the influence of environmental conditions on model agreement and the performance of the analysed GNSS configurations.

### 2.6. Statistical Analysis

The PDOP values and *N*_sat_ assigned to trajectory segments within the analysed areas, as well as for the entire route, were subjected to statistical analysis. For each area, the mean, median, standard deviation (SD), and 95% confidence interval (95% CI) [43,44,45] were calculated for both PDOP and *N*_sat_. These statistics were determined separately for the GPS and GPS/GLONASS configurations, and the differences between them were also computed.

A detailed analysis was conducted of the PDOP and *N*_sat_ differences between the model predictions and observations. For this purpose, data assigned to the same trajectory segments were compared. The distributions of differences were presented in the form of histograms for individual areas and GNSS configurations, enabling the assessment of their variability with respect to observation conditions and the GNSS configuration used. In addition, boxplots were used to compare the distributions, dispersion, and central tendencies of the analysed parameters between the GPS and GPS/GLONASS configurations. The spatial distribution of differences was presented on maps of the analysed areas.

For the comparative assessment of model-observation agreement, the mean absolute error (MAE) and root mean square error (RMSE) were calculated for the PDOP differences. Although traditionally referred to as error metrics, in this study MAE and RMSE quantify discrepancies between model predictions and observations rather than position errors.

The results were interpreted using descriptive statistics, confidence intervals, distribution analyses, mean absolute model-observation differences, statistical significance testing, and effect-size estimates, enabling a quantitative assessment of the influence of the applied GNSS configuration and the degree of model-observation agreement.

To assess whether the absolute model-observation PDOP discrepancies differed significantly between the GPS and GPS/GLONASS configurations, Welch’s independent-samples *t*-test was applied [43,44]. This test was selected because the numbers of trajectory segments retained for analysis differed between the two GNSS configurations and the GPS and GPS/GLONASS datasets could not be uniquely paired on a one-to-one basis; consequently, they were treated as independent samples for the statistical comparison. Welch’s *t*-test is appropriate for unequal sample sizes and does not require the assumption of equal variances.

The null hypothesis (*H*_0_) assumed no difference between the mean |ΔPDOP| values for the GPS and GPS/GLONASS configurations, whereas the alternative hypothesis (*H*_1_) assumed that the means differed. The significance level was set at *α* = 0.05, and the null hypothesis was rejected when *p* < 0.05. To account for the three statistical comparisons performed for the entire route, Kamienny Potok, and Śródmieście, *p*-values were adjusted using the Holm correction [46].

In addition to *p*-values, 95% confidence intervals for the mean differences and Hedges’ *g* effect sizes were calculated. Hedges’ *g* was used as a standardised measure of the magnitude of the difference between the analysed configurations [47]. Statistical significance, confidence intervals, and effect sizes were jointly considered when interpreting differences between the two GNSS configurations.

## 3. Results

### 3.1. Analysis of the Entire Measurement Route

This subsection presents the comparative analysis for the entire measurement route, focusing on the influence of the GPS/GLONASS configuration on PDOP, *N*_sat_, and model-observation agreement. Descriptive statistics of PDOP and *N*_sat_ are summarised in Table 3 and Table 4.

The GPS/GLONASS configuration reduced the mean PDOP value from 2.10 to 1.35 in the model predictions (35.7%) and from 1.81 to 1.27 in the observations (29.8%) relative to the GPS-only configuration. Lower median PDOP values and standard deviations observed for the GPS/GLONASS configuration indicate more favourable and stable satellite constellation geometry than in the GPS-only configuration.

As shown in Table 4, the GPS/GLONASS configuration substantially increased the mean *N*_sat_. The mean *N*_sat_ increased from 8.04 to 15.99 in the model predictions and from 9.74 to 17.82 in the observations, corresponding to increases of approximately 99% and 83%, respectively.

**Figure 4:**
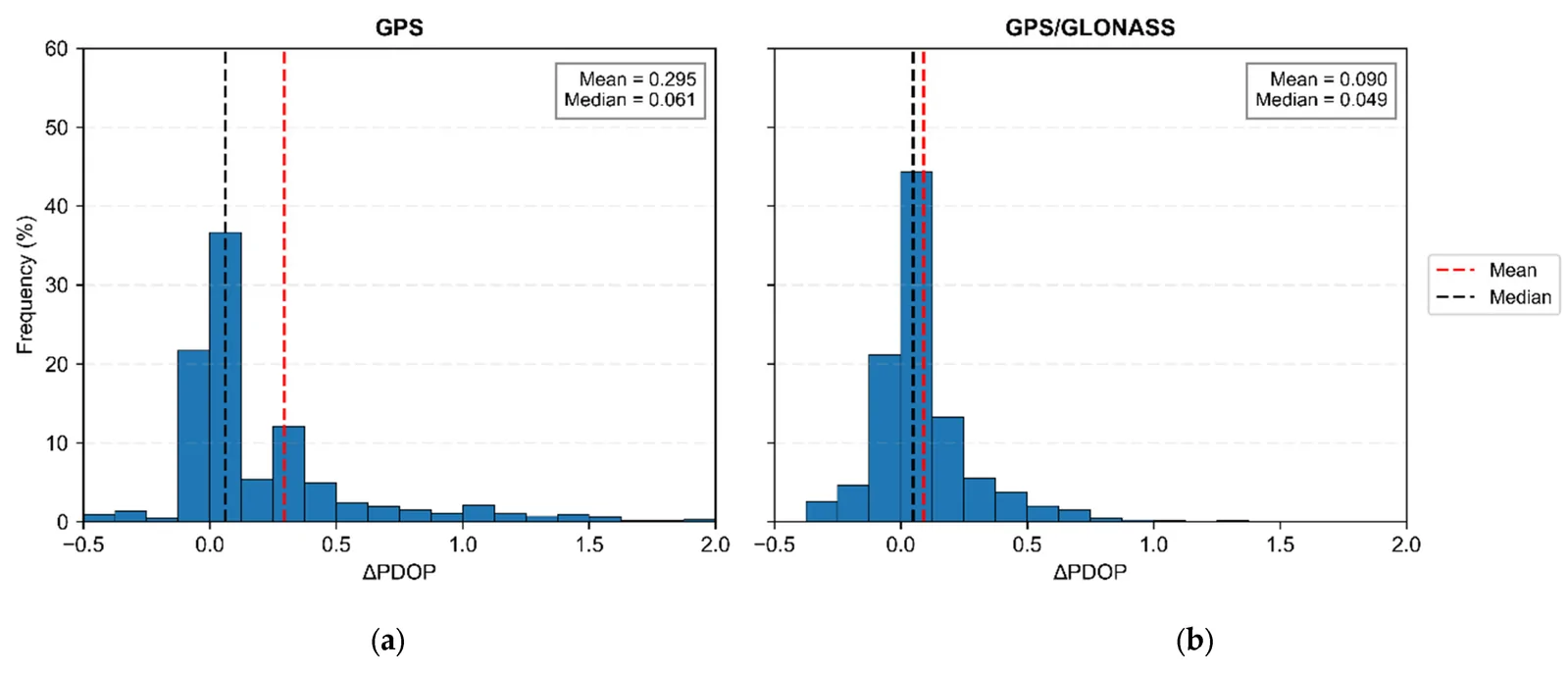
Histograms of ΔPDOP values for GPS (**a**) and GPS/GLONASS (**b**) along the entire measurement route.

Figure 4 presents the histograms of ΔPDOP values for the entire measurement route. For both configurations, most values are concentrated near zero, indicating generally good agreement between the model and the observations. However, the GPS/GLONASS configuration exhibits a noticeably stronger concentration around zero and a narrower spread of values than the GPS-only configuration, confirming better model-observation agreement.

**Figure 5:**
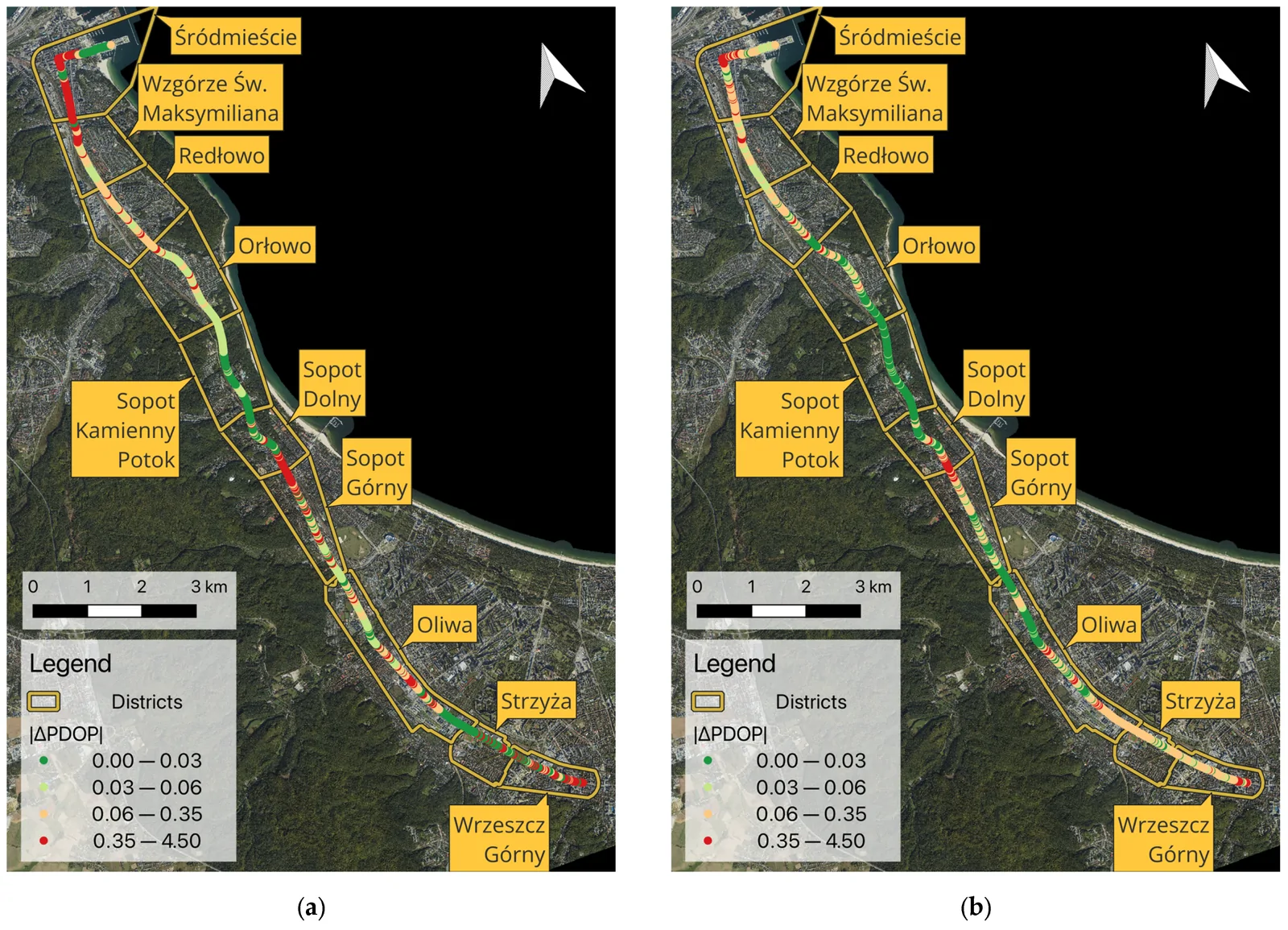
Spatial distribution of |ΔPDOP| between model predictions and observations for GPS (**a**) and GPS/GLONASS (**b**) along the entire measurement route.

Figure 5 presents the spatial distribution of |ΔPDOP| for the GPS and GPS/GLONASS configurations. Higher absolute differences indicate areas of lower model-observation agreement.

The largest discrepancies between model predictions and observations were observed in highly urbanised areas, whereas open areas generally exhibited considerably smaller PDOP differences. The highest |ΔPDOP| values were recorded in the Śródmieście district of Gdynia, whereas the lowest values were observed in the Kamienny Potok district of Sopot. These results indicate that model-observation agreement is strongly dependent on local observation conditions and the degree of GNSS signal obstruction caused by surrounding obstacles.

These results demonstrate that the GPS/GLONASS configuration improves satellite constellation geometry and model-observation agreement under real-world conditions. This is particularly relevant to mobile sensing platforms used in large-scale environmental monitoring, where favourable GNSS observation conditions are important for maintaining reliable spatial referencing of measurements acquired by multiple onboard sensors. Consequently, improved GNSS campaign planning may support more reliable identification of favourable observation conditions along longer measurement routes.

### 3.2. Analysis Under the Most Favourable Observation Conditions

Among the analysed districts of the Tricity area, the district with the most favourable observation conditions was Kamienny Potok in Sopot. This area is characterised by a low level of urbanisation and a substantial proportion of green areas. In addition, the analysed section of the trajectory was located at a higher elevation relative to the surrounding terrain, which reduced potential signal obstructions. As a result, GNSS observations were characterised by consistently high satellite visibility and favourable satellite constellation geometry.

For this area, small differences between the GPS and GPS/GLONASS configurations were observed, along with a high level of model-observation agreement. Descriptive statistics of PDOP and *N*_sat_ are summarised in Table 5 and Table 6.

The mean PDOP value for the GPS/GLONASS configuration decreased from 1.84 to 1.23 in the model predictions (33.2%) and from 1.81 to 1.23 in the observations (32.0%) relative to the GPS-only configuration. These results indicate more favourable and stable satellite constellation geometry for the GPS/GLONASS configuration even under favourable observation conditions.

Only minor differences were observed between the model predictions and observations. For both configurations, the model closely reproduced satellite constellation geometry and visibility, resulting in very small deviations between predictions and measurements.

**Figure 6:**
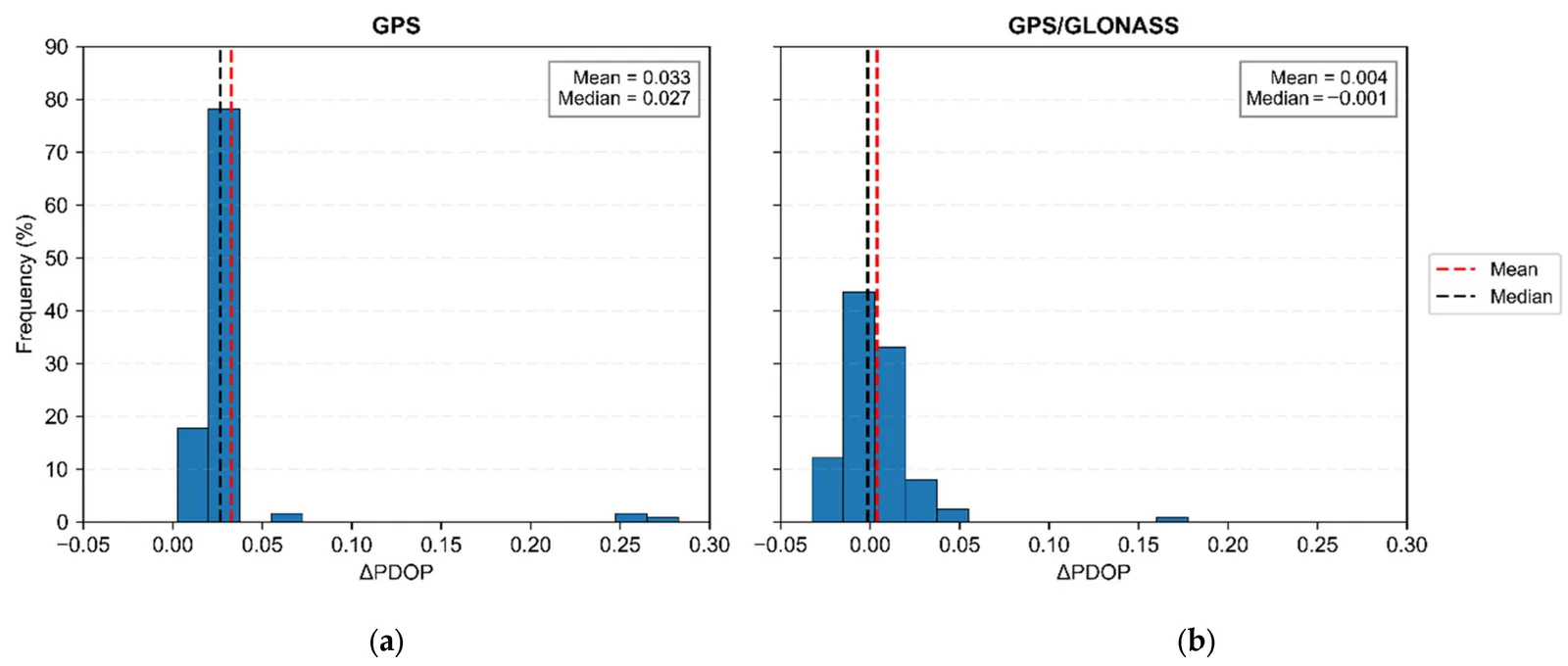
Histograms of ΔPDOP values for GPS (**a**) and GPS/GLONASS (**b**) in the district with the most favourable observation conditions.

Figure 6 presents the histograms of ΔPDOP values for the district with the most favourable observation conditions. For both configurations, the distributions are strongly concentrated around zero, indicating close model-observation agreement. The GPS/GLONASS configuration exhibits a slightly narrower spread of values, indicating marginally better model-observation agreement.

**Figure 7:**
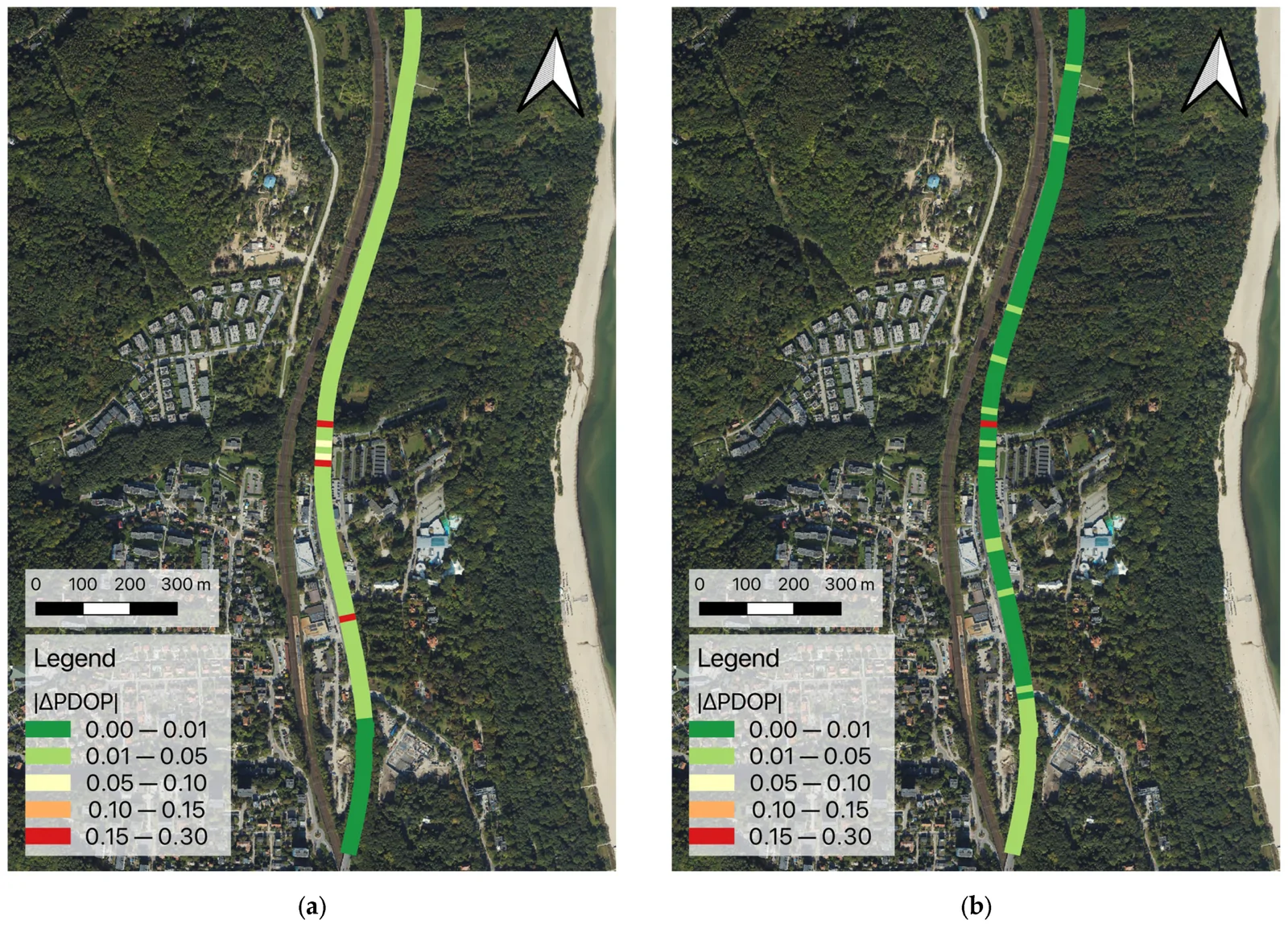
Spatial distribution of |ΔPDOP| between model predictions and observations for GPS (**a**) and GPS/GLONASS (**b**) in the district with the most favourable observation conditions.

Figure 7 presents the spatial distribution of |ΔPDOP| for the GPS and GPS/GLONASS configurations. For the GPS configuration, the spatial distribution of differences is relatively homogeneous, although locally higher values occur in areas with a higher degree of urbanisation.

The GPS/GLONASS configuration resulted in a noticeable reduction in PDOP differences compared with the GPS-only configuration. Higher PDOP differences observed locally may be attributed to inaccuracies in the DSM representation of environmental obstacles. Nevertheless, the overall magnitude of the discrepancies remained low throughout the analysed area.

Under favourable observation conditions, both GNSS configurations demonstrated close model-observation agreement, with consistently lower |ΔPDOP| values for GPS/GLONASS. The greater number of available satellites in the GPS/GLONASS configuration may help maintain favourable satellite constellation geometry along longer measurement routes.

### 3.3. Analysis Under the Most Challenging Observation Conditions

Among the analysed districts of the Tricity area, Śródmieście in Gdynia exhibited the most challenging observation conditions. In this district, the measurement trajectory passed through an environment characterised by dense, compact urban development dominated by high-rise buildings, as well as numerous infrastructure elements, which limited the availability of GNSS signals. These conditions highlighted the differences between the GPS-only and GPS/GLONASS configurations, enabling an assessment of their performance under challenging observation conditions.

For this district, model predictions and observations were compared for both the GPS and GPS/GLONASS configurations. Descriptive statistics of PDOP and *N*_sat_ are summarised in Table 7 and Table 8.

The GPS/GLONASS configuration reduced the mean PDOP value from 2.55 to 1.50 in the model predictions (41.2%) and from 1.80 to 1.27 in the observations (29.4%) relative to the GPS-only configuration. Lower median PDOP values and reduced variability indicate more favourable and stable satellite constellation geometry for the GPS/GLONASS configuration.

As shown in Table 8, the GPS/GLONASS configuration substantially increased the mean *N*_sat_. The mean *N*_sat_ increased from 7.17 to 14.05 in the model predictions and from 9.54 to 17.78 in the observations, corresponding to increases of approximately 96% and 86%, respectively.

**Figure 8:**
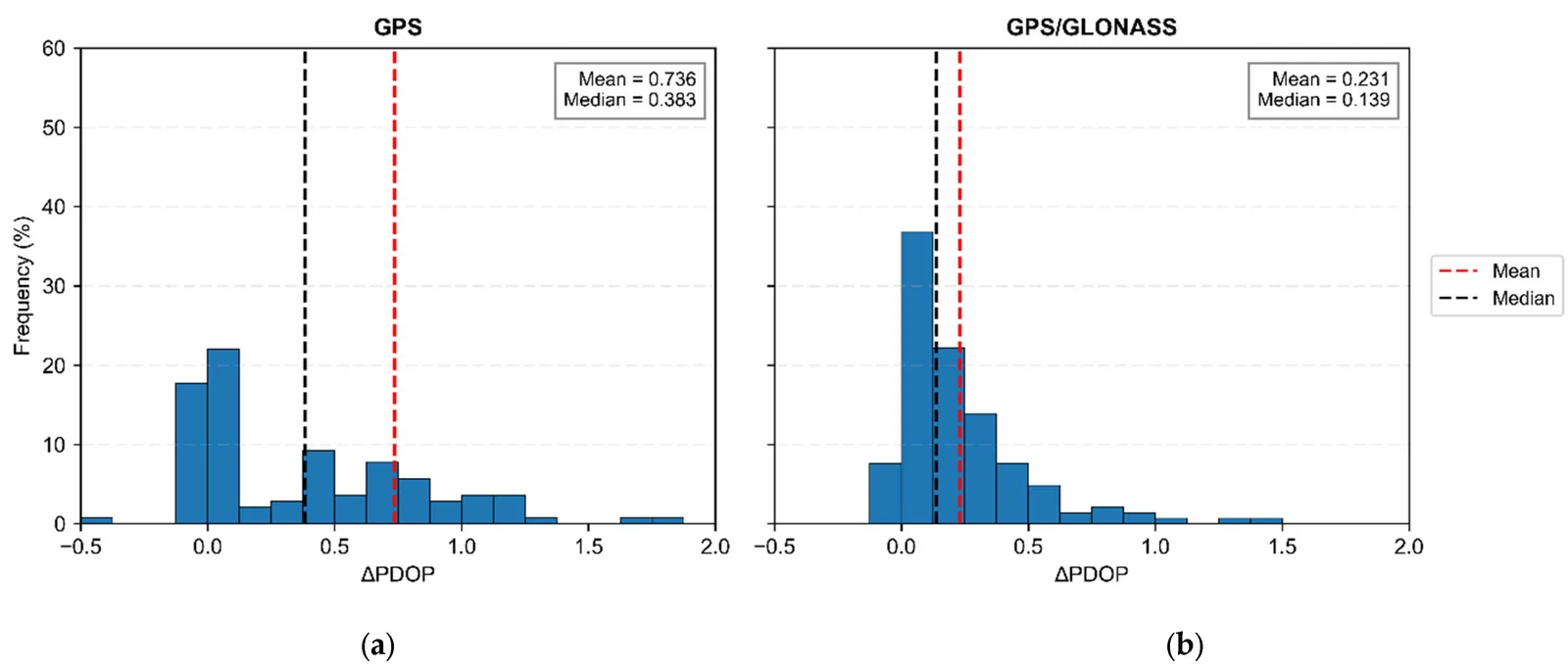
Histograms of ΔPDOP values for GPS (**a**) and GPS/GLONASS (**b**) in the district with the most challenging observation conditions.

Figure 8 presents the histograms of ΔPDOP values for the district with the most challenging observation conditions. The distributions reveal a clear difference between the analysed configurations. The histogram for the GPS configuration exhibits a substantially wider spread, whereas the GPS/GLONASS configuration is more strongly concentrated around zero, confirming better model-observation agreement.

A comparison of the model predictions with the measurement data indicates considerably larger deviations for the GPS configuration. The positive ΔPDOP values indicate a tendency of the model to overestimate PDOP under challenging observation conditions.

**Figure 9:**
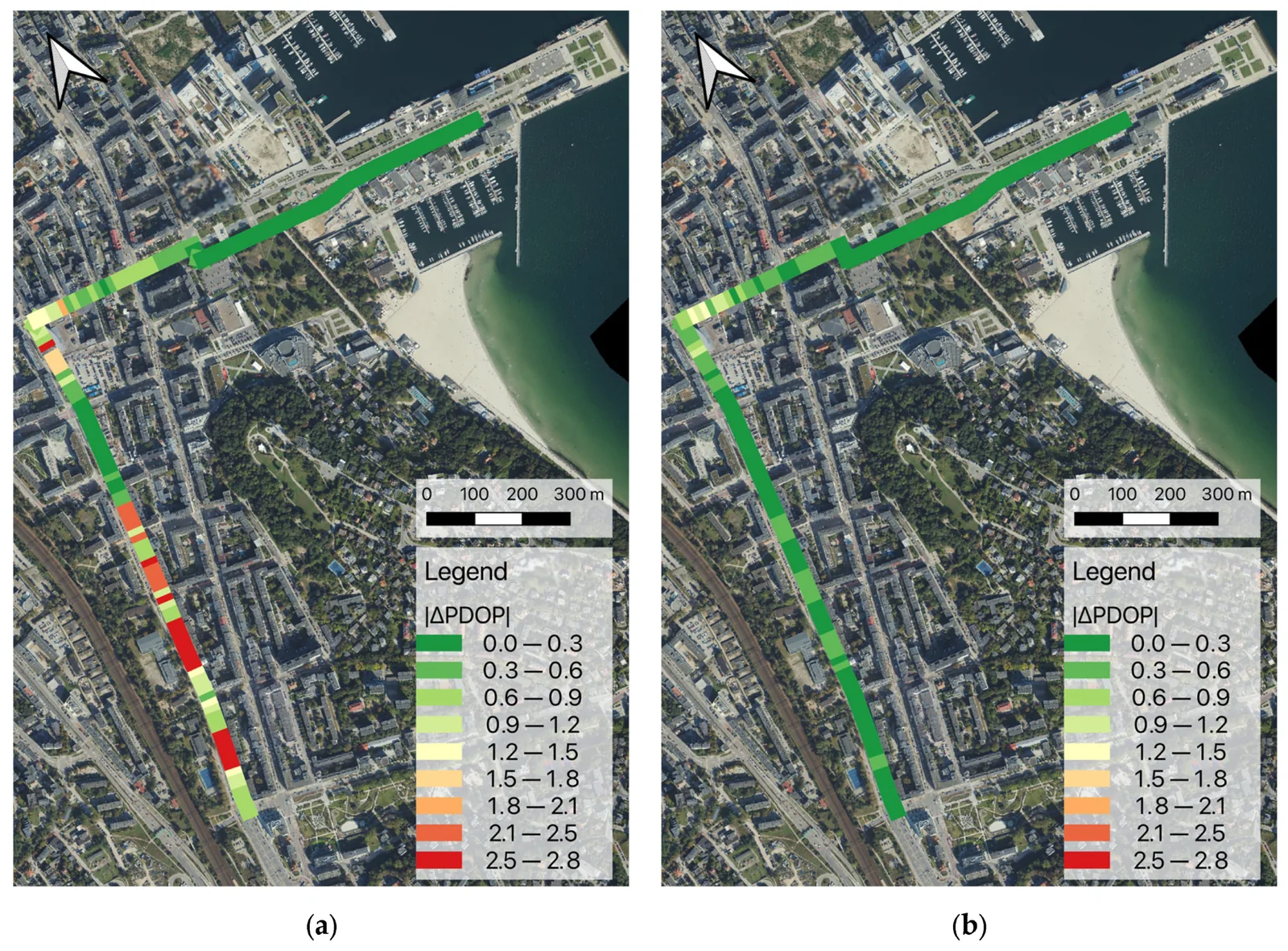
Spatial distribution of |ΔPDOP| between model predictions and observations for GPS (**a**) and GPS/GLONASS (**b**) in the district with the most challenging observation conditions.

The GPS/GLONASS configuration achieved better model-observation agreement than the GPS-only configuration. Although the model underestimated the mean *N*_sat_ by approximately four satellites, the differences in PDOP values and their variability remained substantially smaller than those observed for the GPS configuration. Figure 9 presents the spatial distribution of |ΔPDOP| for the analysed district.

For the GPS configuration, the differences are clearly spatially variable. The largest discrepancies occur in the highly urbanised part of the district, whereas the smallest are observed in more open areas, including the vicinity of Molo Południowe.

The GPS/GLONASS configuration resulted in a noticeable reduction in these model-observation differences, particularly in the southern, highly urbanised part of the district. Higher discrepancies are still observed in the central part of the district, where dense urban development, tall buildings, and narrow street corridors create the most challenging GNSS observation conditions.

The improvements observed for the GPS/GLONASS configuration are particularly relevant to sensor-based environmental monitoring conducted in complex urban environments. In such areas, favourable satellite constellation geometry is an important prerequisite for reliable GNSS-supported georeferencing of measurements acquired by optical, laser, hydrographic, and other sensors mounted on mobile platforms. The results therefore indicate that the GPS/GLONASS configuration can improve satellite constellation geometry and provide closer model-observation agreement under challenging urban conditions.

### 3.4. Comparative Summary of the GPS and GPS/GLONASS Configurations

Building on the results presented in Section 3.1, Section 3.2 and Section 3.3, this section provides a comparison of the GPS and GPS/GLONASS configurations in terms of model-observation agreement. The comparison is based on the distributions and spatial patterns of |ΔPDOP|, together with a quantitative statistical assessment using the trajectory segments retained separately for each GNSS configuration.

**Figure 10:**
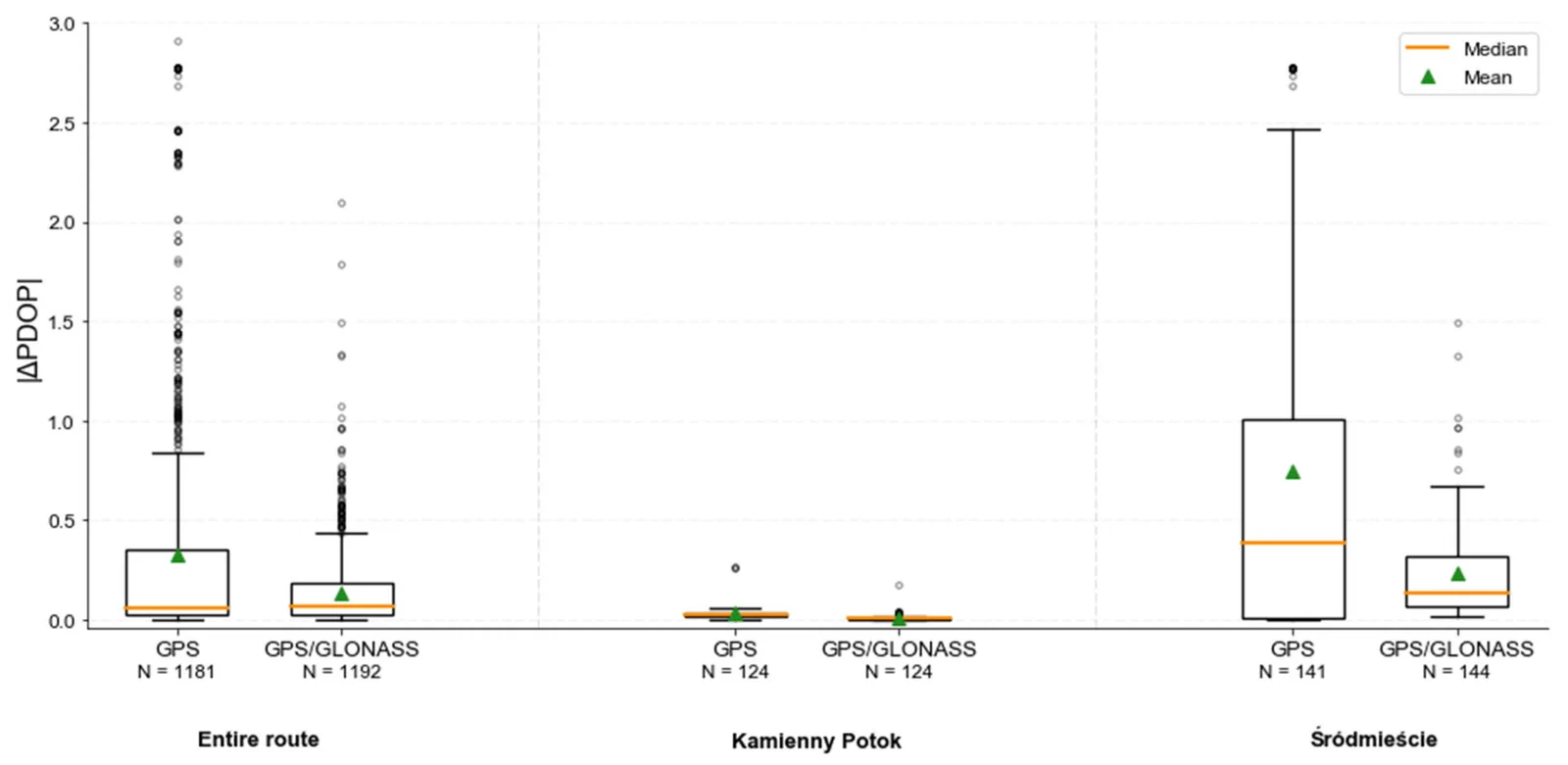
Boxplots of absolute model-observation PDOP differences (|ΔPDOP|) for the GPS and GPS/GLONASS configurations along the entire measurement route and in two representative districts: Kamienny Potok, representing the most favourable observation conditions, and Śródmieście, representing the most challenging observation conditions. *N* denotes the number of trajectory segments retained for analysis in each GNSS configuration.

Figure 10 compares the distributions of |ΔPDOP| obtained for the GPS and GPS/GLONASS configurations along the entire measurement route and within the districts representing the most favourable and the most challenging observation conditions. Across all analysed scenarios, the GPS/GLONASS configuration exhibits a more compact distribution of |ΔPDOP|, characterised by lower dispersion and substantially fewer large discrepancies than the GPS-only configuration. Although the median |ΔPDOP| value for the entire route is slightly higher for the GPS/GLONASS configuration, its lower spread and shorter upper tail indicate more consistent overall model-observation agreement.

The absolute reduction in model-observation discrepancies was substantially greater under challenging observation conditions, whereas the relative MAE reductions were similar in the two representative environments. Under favourable observation conditions, both configurations exhibit similarly small discrepancies, whereas under challenging urban conditions the GPS/GLONASS configuration substantially reduces both the magnitude and variability of the model-observation discrepancies.

**Figure 11:**
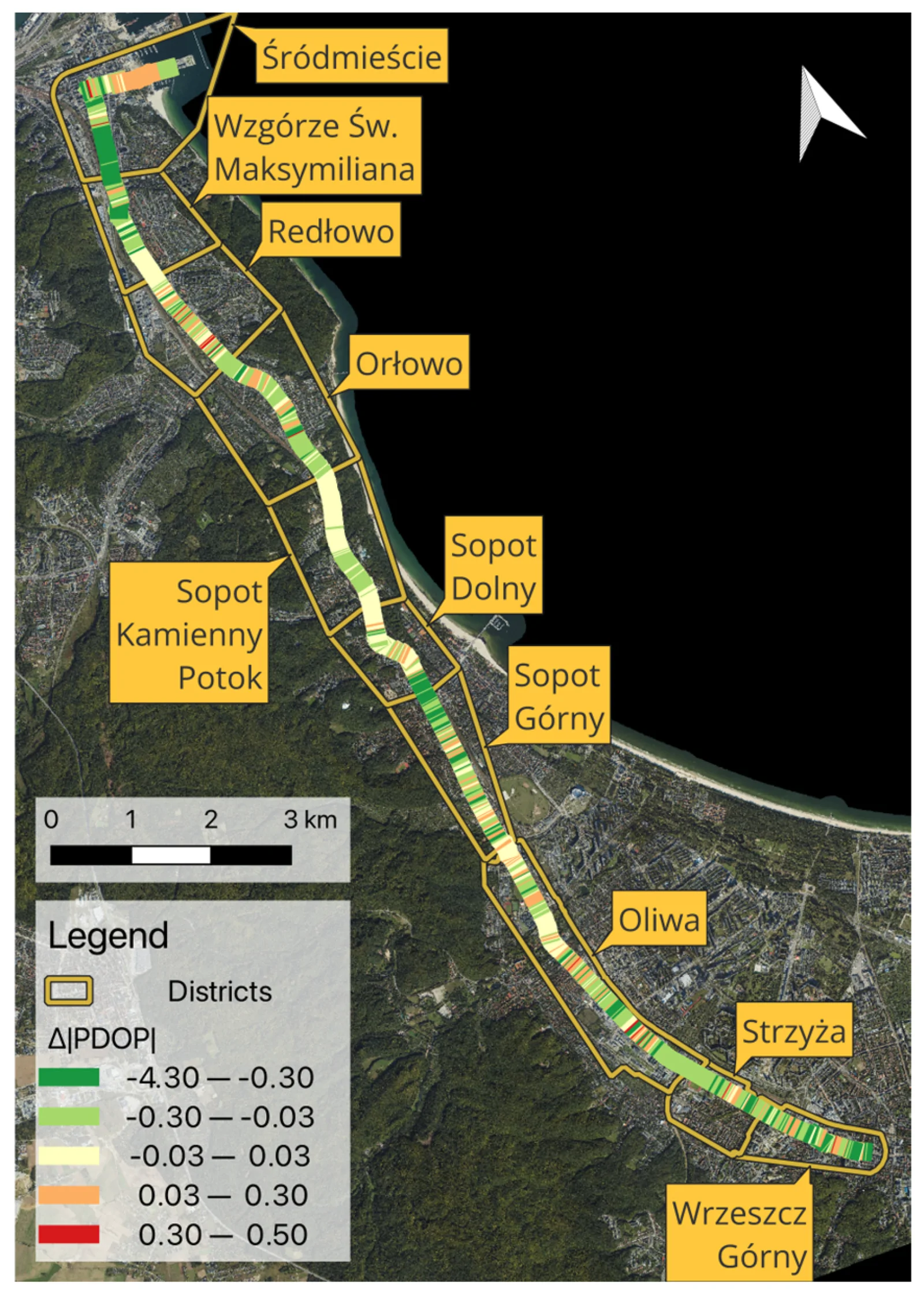
Spatial distribution of Δ|PDOP| between the GPS and GPS/GLONASS configurations along the measurement route.

Figure 11 illustrates the spatial distribution of Δ|PDOP| along the measurement route, highlighting the differences in model-observation agreement between the GPS and GPS/GLONASS configurations. Negative values of Δ|PDOP| indicate trajectory segments where the GPS/GLONASS configuration provides closer agreement with the field observations, whereas positive values indicate trajectory segments where the GPS-only configuration provides slightly closer agreement.

The spatial distribution of Δ|PDOP| confirms that the benefits of extending the configuration from GPS-only to GPS/GLONASS are not uniform along the measurement route. The largest absolute improvements are concentrated in the district representing the most challenging observation conditions, where surrounding buildings substantially restrict satellite visibility and degrade satellite constellation geometry. In contrast, Δ|PDOP| values close to zero predominate in the district representing the most favourable observation conditions, indicating that both configurations provide comparable model-observation agreement in areas with largely unobstructed sky visibility. Only isolated trajectory segments exhibit positive values of Δ|PDOP|, suggesting a slight local advantage of the GPS-only configuration; however, these cases are infrequent and do not influence the overall spatial distribution. Overall, these results demonstrate that the GPS/GLONASS configuration provides more consistent model-observation agreement, particularly in environments characterised by limited satellite visibility and significant signal obstructions.

The absolute model-observation PDOP discrepancies were further compared between the GPS and GPS/GLONASS configurations using Welch’s independent-samples *t*-test. Statistically significant differences were found for the entire route (*t* = 10.590, degrees of freedom (*df*) = 1419.84, *p* < 0.001), Kamienny Potok (*t* = 5.826, *df* = 175.55, *p* < 0.001), and Śródmieście (*t* = 6.253, *df* = 160.13, *p* < 0.001). All differences remained statistically significant after Holm correction for multiple comparisons. In all three cases, the mean |ΔPDOP| was lower for the GPS/GLONASS configuration.

The corresponding Hedges’ *g* effect sizes were 0.436, 0.738, and 0.746 for the entire route, Kamienny Potok, and Śródmieście, respectively. The effect size was smaller for the entire route than for either representative district. In all three comparisons, the 95% confidence intervals for the mean differences remained entirely above zero. The results of the statistical comparison are summarised in Table 9.

These results demonstrate that the reduction in model-observation discrepancies is supported not only by descriptive statistics but also by statistical significance testing and effect-size analysis. The statistical comparison confirms systematic differences between the GPS and GPS/GLONASS configurations, while the effect-size analysis indicates that the magnitude of these differences varies between the entire route and the representative observation environments.

## 4. Discussion

This study demonstrates that extending the constellation configuration from GPS-only to GPS/GLONASS improves agreement between model predictions and field observations. Improvements were observed along the entire measurement route and under both favourable and challenging observation conditions.

The principal contribution of the present study should be distinguished from the well-established fact that adding GNSS constellations generally increases satellite availability and reduces DOP values. The relevant result is that constellation configuration also affects the agreement between trajectory-based campaign-planning predictions and field observations. Moreover, the magnitude of this effect is spatially variable and depends on the local observation environment. This represents a different validation objective from the preceding GPS-only study [40], which focused primarily on identifying the sources of model-observation discrepancies.

As shown in Table 10, the GPS/GLONASS configuration consistently reduced MAE, RMSE, standard deviation, and the 95th percentile relative to the GPS-only configuration. Particularly large decreases in RMSE and the 95th percentile indicate that the GPS/GLONASS configuration reduced both the overall magnitude of model-observation discrepancies and the occurrence of large discrepancies, especially under challenging urban conditions.

To facilitate comparison between the analysed environments, the percentage reduction in MAE obtained using the GPS/GLONASS configuration relative to the GPS-only configuration is summarised in Table 11.

The largest relative MAE reduction was observed under the most challenging conditions (68.4%), compared with 66.7% under favourable conditions and 58.0% for the entire route. These results show substantial reductions in model-observation discrepancies in both representative environments, with the largest absolute discrepancies and absolute improvement observed under challenging urban conditions.

The results of Welch’s *t*-test support the patterns observed in the descriptive statistics. Differences in |ΔPDOP| between the GPS and GPS/GLONASS configurations were statistically significant in all three analysed areas (*p* < 0.001) and remained significant after Holm correction for multiple comparisons. Hedges’ *g* was 0.436 for the entire route, 0.738 for Kamienny Potok, and 0.746 for Śródmieście, indicating that the magnitude of the difference was greater in the two representative districts than for the entire route. In all three comparisons, the 95% confidence intervals for the mean differences remained above zero, consistent with lower mean |ΔPDOP| values for the GPS/GLONASS configuration.

The statistical results therefore provide additional support for the reduction in model-observation PDOP discrepancies observed for the GPS/GLONASS configuration. Considering the *p*-values together with the confidence intervals and Hedges’ *g* effect sizes provides a more comprehensive assessment of the differences between the two GNSS configurations than statistical significance alone.

The magnitude of the improvement should be interpreted in relation to the observation environment rather than solely as a consequence of the larger number of available satellites. Under favourable conditions, the GPS-only configuration already provides relatively high satellite availability and stable constellation geometry, leaving limited scope for substantial absolute improvement. In contrast, under challenging urban conditions, the obstruction of signals from individual satellites can have a greater influence on the geometry of the remaining constellation. The additional GLONASS satellites increase satellite availability and can help maintain more favourable constellation geometry despite local signal obstructions. This mechanism may contribute to the substantial reduction in model-observation discrepancies observed under challenging urban conditions.

Although the GPS/GLONASS configuration substantially improved model-observation agreement, residual discrepancies remained throughout the analysed route. These discrepancies arise primarily from limitations of the digital surface model used to represent environmental obstacles and from the simplified modelling of GNSS signal propagation. In particular, the model does not explicitly account for multipath propagation or NLOS signals, both of which are common in urban environments and may significantly affect satellite visibility and positioning quality. Furthermore, PDOP describes only the geometric component of GNSS positioning quality and does not account for position errors caused by multipath propagation, receiver characteristics, or atmospheric effects. Consequently, agreement in terms of PDOP should be interpreted as agreement in satellite constellation geometry rather than overall positioning accuracy. Accordingly, the present validation concerns the predictive capability of the model with respect to satellite visibility and satellite constellation geometry and should not be interpreted as a validation of positioning accuracy itself.

Conventional GNSS campaign-planning tools generally evaluate satellite availability and DOP at predefined stationary locations using fixed elevation masks or simplified obstruction assumptions. In contrast, the model evaluated in this study was specifically developed for measurements conducted in motion along linear features and integrates trajectory-dependent satellite constellation geometry with DSM/DTM-based obstruction modelling. Consequently, a direct numerical comparison with conventional point-based planning tools would not constitute a like-for-like validation of the trajectory-based functionality considered here. The primary validation approach therefore consisted of comparison with real kinematic field measurements. Nevertheless, systematic benchmarking against other trajectory-aware GNSS planning approaches, where comparable functionality is available, constitutes an important direction for future research.

The purpose of the planning model is not merely to indicate that dense urban environments generally provide less favourable GNSS conditions. Such a qualitative relationship is already well established. Its practical purpose is to predict, before field deployment, where and when along a specific measurement trajectory satellite constellation geometry may become unfavourable and to support the selection of an appropriate campaign schedule. The spatial variability observed within individual districts further demonstrates that observation quality cannot be inferred solely from a general classification of an area as urban or open.

The present study has several limitations. The analysis was based on a single measurement campaign conducted using one receiver model and was limited to GPS and GPS/GLONASS observations. However, it represents one stage within a sequential field-validation framework. The first stage considered the GPS-only configuration and focused on identifying the principal sources of model-observation discrepancies. The present stage evaluates the effect of extending the constellation configuration from GPS-only to GPS/GLONASS while retaining comparable measurement conditions. A subsequent stage will extend the analysis to full multi-GNSS configurations incorporating GPS, GLONASS, Galileo, and BeiDou using two geodetic-grade multi-GNSS receivers and a higher GNSS sampling frequency. This staged design enables the influence of successive changes in the observation configuration to be evaluated rather than combining all system extensions within a single experiment. Future studies should include repeated measurement campaigns conducted under different environmental and seasonal conditions and additional receiver types. Further research may also investigate other DOP indicators and more advanced models of urban signal propagation to improve the predictive capability of the proposed planning methodology.

From a practical perspective, the proposed methodology supports survey planning based on the expected quality of satellite constellation geometry before field deployment. By identifying route sections characterised by potentially unfavourable observation conditions, operators can optimise measurement schedules and minimise the risk of degraded positioning geometry during data acquisition. Accordingly, the GPS/GLONASS configuration may be preferable to the GPS-only configuration for campaign planning in urban and partially obstructed environments.

Beyond validating the GNSS campaign planning model, the results have broader implications for sensor-based environmental monitoring applications employing mobile sensing platforms. Reliable GNSS campaign planning can support the identification of observation periods and route sections characterised by favourable satellite constellation geometry, which is an important prerequisite for GNSS-supported georeferencing of measurements acquired by optical, laser, hydrographic, and environmental sensors. These findings are consistent with previous studies highlighting the importance of GNSS-based spatial referencing for multisensor environmental monitoring, mobile mapping systems, and unmanned sensing platforms [1,5,8,9,12]. Consequently, the proposed methodology may support the planning of measurement campaigns involving UAVs, USVs, mobile mapping systems, infrastructure inspection, and other mobile environmental monitoring applications operating in complex environments.

## 5. Conclusions

This study has presented a quantitative validation of a GNSS campaign planning model and demonstrated the benefits of a GPS/GLONASS configuration for GNSS campaign planning in kinematic surveys under spatially varying GNSS observation conditions.

The results lead to the following conclusions:

1. The GPS/GLONASS configuration substantially improved satellite constellation geometry relative to the GPS-only configuration. Mean PDOP values decreased by approximately 30% in the observations and by 33–41% in the model predictions;
2. The GPS/GLONASS configuration substantially increased satellite availability. The mean *N*_sat_ increased by approximately 81–86% in the observations and by approximately 96–100% in the model predictions;
3. The GPS/GLONASS configuration improved model-observation agreement across all analysed environments. The mean absolute model-observation PDOP difference was reduced by 58.0% for the entire route, 66.7% under favourable observation conditions, and 68.4% under challenging observation conditions;
4. The influence of extending the constellation configuration from GPS-only to GPS/GLONASS was environment-dependent, with the largest absolute improvement observed under challenging urban conditions characterised by substantial signal obstruction;
5. The proposed planning model provides a framework for identifying favourable GNSS observation conditions and supporting the planning of kinematic measurement campaigns.

Despite the identified limitations, the results confirm the potential of the GPS/GLONASS configuration to improve satellite constellation geometry and model-observation agreement in GNSS campaign planning for kinematic surveys. Future research should include additional measurement campaigns, different receiver types, and validation of the proposed approach in practical sensor-based environmental monitoring applications employing UAVs, USVs, and terrestrial mobile mapping systems under diverse environmental conditions.

The present results should therefore be interpreted as a controlled validation of the transition from a single-constellation GPS configuration to a dual-constellation GPS/GLONASS configuration rather than as a comprehensive evaluation of full multi-GNSS performance. Validation incorporating Galileo and BeiDou using geodetic-grade receivers represents the next planned stage of the research.

## Figures and Tables

**Table 1:** Comparison between the previous GPS-based validation study and the present study.

Aspect	Previous Study	Present Study
Main objective	Validation of the GNSS campaign planning model and identification of the principal sources of model-observation discrepancies	Assessment of constellation configuration as a factor affecting model-observation agreement
GNSS configurations analysed	GPS	GPS and GPS/GLONASS
Satellite visibility analysis	GPS-only	GPS vs. GPS/GLONASS
PDOP analysis	GPS-only	GPS vs. GPS/GLONASS
Comparison of GPS and GPS/GLONASS configurations	No	Yes
Analysis of discrepancies between model predictions and observations	GPS-only	GPS vs. GPS/GLONASS
Quantitative assessment of model-observation agreement	GPS-only assessment	Comparative GPS vs. GPS/GLONASS assessment across different observation environments
Observation environments analysed	Entire route	Entire route and representative favourable and challenging observation environments
Statistical analysis	Basic descriptive statistics	Descriptive statistics, Welch’s *t*-test, confidence intervals, and effect-size analysis
Main outcome	Validation of the planning model	Quantitative assessment of how extending the configuration from GPS-only to GPS/GLONASS affects model-observation agreement under different observation conditions

**Table 2:** Model parameters for GNSS campaign planning (based on [20]).

Parameter	Value	Remarks
Date	10 September 2024	
Start time	22:00:00	Night-time measurements (reduced traffic)
Time window	3 h	
Vehicle speed	50 km/h	
Recording interval	1 s	
Elevation mask	10°	
Maximum PDOP value	7	Geometry quality threshold
Antenna height	1.7 m	
DOP metric used	PDOP	
Planning variant	GPS; GPS/GLONASS	Comparative analysis
GPS orbital data	YUMA almanac	GPS week 2329 (Time of Applicability, ToA = 589,824 s)
GLONASS orbital data	GLONASS NAV DATA, version 2.01	IGS broadcast ephemeris file brdc2540.24g; CDDIS, day 254 of 2024
Minimum stop duration	5 min	
Minimum distance between stops	1 km	
Route	Gdańsk-Sopot-Gdynia	Linear feature
DSM	Geoportal	0.5 m, 2022, PL-EVRF2007-NH
DTM	Geoportal	0.5 m, 2022, PL-EVRF2007-NH

**Table 3:** Descriptive statistics of PDOP values along the entire measurement route.

GNSS Configuration	Data Source	*N*	Mean	Median	SD	95% CI
GPS	Model	1308	2.10	1.83	0.60	2.06–2.13
GPS	Observations	1195	1.81	1.81	0.11	1.80–1.81
GPS/GLONASS	Model	1322	1.35	1.28	0.20	1.34–1.36
GPS/GLONASS	Observations	1192	1.27	1.24	0.07	1.26–1.27

Note: *N* denotes the number of trajectory segments retained for analysis. PDOP is dimensionless.

**Table 4:** Descriptive statistics of *N*_sat_ along the entire measurement route.

GNSS Configuration	Data Source	*N*	Mean	Median	SD	95% CI
GPS	Model	1308	8.04	9.00	1.24	7.97–8.11
GPS	Observations	1195	9.74	10.00	0.52	9.71–9.77
GPS/GLONASS	Model	1322	15.99	17.00	2.32	15.87–16.12
GPS/GLONASS	Observations	1192	17.82	18.00	0.65	17.79–17.86

Note: *N* denotes the number of trajectory segments retained for analysis; *N*_sat_ denotes the number of visible satellites.

**Table 5:** Descriptive statistics of PDOP values for the district with the most favourable observation conditions.

GNSS Configuration	Data Source	*N*	Mean	Median	SD	95% CI
GPS	Model	127	1.84	1.83	0.04	1.83–1.84
GPS	Observations	124	1.81	1.81	0.01	1.80–1.81
GPS/GLONASS	Model	127	1.23	1.23	0.02	1.23
GPS/GLONASS	Observations	124	1.23	1.23	0.01	1.23

Note: *N* denotes the number of trajectory segments retained for analysis. PDOP is dimensionless.

**Table 6:** Descriptive statistics of *N*_sat_ for the district with the most favourable observation conditions.

GNSS Configuration	Data Source	*N*	Mean	Median	SD	95% CI
GPS	Model	127	8.96	9.00	0.19	8.93–8.99
GPS	Observations	124	10.00	10.00	0.00	10.00
GPS/GLONASS	Model	127	17.95	18.00	0.30	17.90–18.01
GPS/GLONASS	Observations	124	18.09	18.00	0.28	18.04–18.14

Note: *N* denotes the number of trajectory segments retained for analysis; *N*_sat_ denotes the number of visible satellites.

**Table 7:** Descriptive statistics of PDOP values for the district with the most challenging observation conditions.

GNSS Configuration	Data Source	*N*	Mean	Median	SD	95% CI
GPS	Model	155	2.55	2.21	0.99	2.40–2.71
GPS	Observations	147	1.80	1.73	0.13	1.78–1.82
GPS/GLONASS	Model	161	1.50	1.42	0.26	1.46–1.54
GPS/GLONASS	Observations	144	1.27	1.27	0.05	1.26–1.28

Note: *N* denotes the number of trajectory segments retained for analysis. PDOP is dimensionless.

**Table 8:** Descriptive statistics of *N*_sat_ for the district with the most challenging observation conditions.

GNSS Configuration	Data Source	*N*	Mean	Median	SD	95% CI
GPS	Model	155	7.17	7.00	1.71	6.90–7.44
GPS	Observations	147	9.54	10.00	0.55	9.45–9.63
GPS/GLONASS	Model	161	14.05	15.00	2.63	13.64–14.46
GPS/GLONASS	Observations	144	17.78	18.00	0.74	17.66–17.90

Note: *N* denotes the number of trajectory segments retained for analysis; *N*_sat_ denotes the number of visible satellites.

**Table 9:** Statistical comparison of absolute model-observation PDOP discrepancies between the GPS and GPS/GLONASS configurations.

Study Area	*N* (GPS/GPS/GLONASS)	Mean Difference (GPS − GPS/GLONASS)	*t*	*df*	Adjusted *p*	95% CI for Mean Difference	Hedges’ *g*
Entire route	1181/1192	0.188	10.590	1419.84	<0.001	0.153–0.223	0.436
Kamienny Potok	124/124	0.022	5.826	175.55	<0.001	0.0145–0.0295	0.738
Śródmieście	141/144	0.512	6.253	160.13	<0.001	0.350–0.674	0.746

Note: The mean difference was calculated as the mean |ΔPDOP| for the GPS configuration minus that for the GPS/GLONASS configuration. Positive values therefore indicate lower mean model-observation discrepancies for the GPS/GLONASS configuration. *N* denotes the number of trajectory segments retained for each configuration. Adjusted *p*-values were obtained using the Holm correction.

**Table 10:** Descriptive statistics of |ΔPDOP| for the GPS and GPS/GLONASS configurations.

Study Area	GNSS Configuration	*N*	MAE	RMSE	Median	SD	95th Percentile
Entire route	GPS	1181	0.324	0.665	0.059	0.581	1.473
	GPS/GLONASS	1192	0.136	0.231	0.07	0.187	0.482
Kamienny Potok	GPS	124	0.033	0.05	0.027	0.038	0.037
	GPS/GLONASS	124	0.011	0.021	0.007	0.018	0.034
Śródmieście	GPS	141	0.749	1.198	0.383	0.939	2.776
	GPS/GLONASS	144	0.237	0.347	0.139	0.255	0.745

Note: *N* denotes the number of trajectory segments retained for analysis. All quantities reported in the table are dimensionless.

**Table 11:** Reduction in model-observation PDOP MAE for the GPS/GLONASS configuration relative to the GPS-only configuration.

Study Area	${\left|\bar{\Delta PDOP}\right|}_{GPS}$ (–)	${\left|\bar{\Delta PDOP}\right|}_{GPS/GLONASS}$	Reduction (%)
Entire route	0.324	0.136	58.0
Kamienny Potok	0.033	0.011	66.7
Śródmieście	0.749	0.237	68.4

## Data Availability

The original contributions presented in the study are included in the article; further inquiries can be directed to the corresponding author.
